# A Unique Case of Poorly Differentiated Neuroendocrine Carcinoma With Brain and Bilateral Adrenal Metastases

**DOI:** 10.1002/cnr2.70130

**Published:** 2025-02-02

**Authors:** Carla J. El Hajj Mouawad, Francois Georges Kamar

**Affiliations:** ^1^ Pharmacy Doctor Saint Joseph University of Beirut: Faculty of Pharmacy, BCPS (Board certified pharmacotherapy specialist) Beirut Lebanon; ^2^ Medical Doctor, Associate Professor of Medicine University of Balamand‐ School of Medicine & Medical Sciences. Hematology‐Oncology & Neuro‐Oncology. Head & Program Director of the hematology‐Oncology Division, Mount Lebanon Hospital Hazmieh Lebanon

**Keywords:** bilateral adrenal metastases, high grade, neuroendocrine carcinoma, paraneoplastic syndrome, small cell

## Abstract

**Background:**

Small‐cell carcinoma is a highly malignant neuroendocrine neoplasm arising from cells of the endocrine and nervous systems, and usually of bronchogenic origin. When found in the retroperitoneum, these malignant cells are extremely rare and are mainly metastatic tumors. Adrenal glands are unusual sites of distant metastases, the common primary being bronchopulmonary and gastroenteropancreatic neuroendocrine tumors (NETs). We report a case of poorly differentiated neuroendocrine carcinoma (NEC) that was initially discovered in both adrenal glands.

**Case:**

Our patient was a 68‐year‐old woman who presented with articular pain and severe chronic hemolytic anemia. Her workup comprised a contrast‐enhanced computed tomography (CT) scan of the chest, abdomen, and pelvis, revealing a left adrenal mass lesion measuring 14 × 9 cm, and a concomitantly smaller right adrenal mass lesion arising from the gland and measuring 4 × 2 cm. In view of the size of the left adrenal mass we elected to offer her a complete resection. The patient therefore underwent a laparoscopic adrenalectomy. Histopathological examination of the specimen revealed a high‐grade, poorly differentiated NEC of the adrenal gland, small‐cell type, with tumor necrosis. A baseline evaluation comprised an FDG‐PET CT scan revealing the contralateral adrenal tumor, which was also partially resected to leave the patient with some functional adrenal tissue and not render her Addisonian. Although we found no pulmonary primary, a bleb was seen on chest CT that we hypothesized was possibly a burn‐out primary in the setting of an immunogenic tumor.

**Conclusion:**

Surgery played a vital role in our case followed by combination of chemoimmunotherapy as per present recommendation of a small‐cell tumor especially since the patient presented with atypical clinical manifestations.

## 1 Introduction

This case represents the rare finding of a small‐cell neuroendocrine carcinoma (SCNEC) confined to both adrenal glands where the primary of the tumor could not be located. The patient also developed brain metastases which are uncommon in NEC originating from the genitourinary system. In addition to a dual paraneoplastic syndrome (PNS) characterized with myopathy, algodystrophy and hemolytic anemia.

Primary small‐cell carcinoma of the adrenal gland is an extremely rare malignancy. Some tumors are functional and symptomatic, whereas others are nonfunctional and found incidentally. The overall incidence of high‐grade neuroendocrine tumor (NET) is estimated to be 4.44% in Caucasians [1].

We present a case of SCNEC of both adrenal glands that was incidentally discovered during anemia workup and successfully treated with surgery, adjuvant chemotherapy, and radiotherapy.

## 2 Case Presentation

**FIGURE 1:**
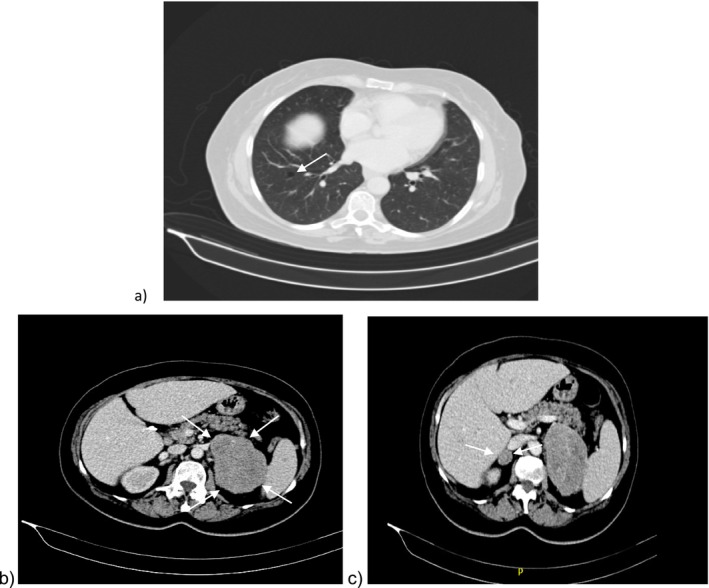
Initial computed tomography (CT) of the abdomen/pelvis. (a) 7 mm bleb in the right lower lobe most probably the origin of the tumor. (b) Large, heterogenous retroperitoneal mass arising from the left adrenal gland, measuring 14 × 9 cm in axial representation. (c) Large, heterogenous retroperitoneal mass arising from the left adrenal gland, measuring 14 × 9 cm and a controlateral 2 cm tumor arising from the right adrenal in axial representation. No magnification of any of the images was done. They were copied as they appeared on the viewer.

The patient is a 68‐year‐old female who presented to Mount Lebanon hospital in February 2022 for a complete workup of hemolytic normocytic normochromic anemia, Hb: 7.2 g/dL [12.0–15.0 g/dL] of nearly 4 months. The patient was unable to walk and reported several months of excruciating articular pain mainly in the lower extremities, 12 kg weight loss over the past 6 months, and had rheumatological workup. She had no significant medical history except for hypertension and mild hypercholesterolemia. She had been an ex‐smoker for 30 years. The anemia workup included protein electrophoresis and bone marrow biopsy, all of which were normal. A contrast‐enhanced total body computed tomography (CT) scan was then performed to rule out hepato‐splenomegaly and has led to the incidental finding of a 14 × 9 cm mass arising from the left adrenal gland with extensive necrosis (Figure 1).

Endocrine workup was performed and indicated a metabolically inactive tumor that was discovered incidentally, with cortisol, aldosterone, VMA, 5‐HIAA, metanephrine, and amphetamine levels measured in 24 urine collections. All tests returned with normal results.

As far as the management of the disease is concerned, surgery played a vital role [2, 3, 4]. Given the potentially resectable tumor according to preoperative imaging, surgery was hence indicated, and the patient underwent laparoscopic radical left adrenalectomy. Pathological examination revealed a completely resected SCNEC with negative margins. No lympho‐vascular nor perineural invasion was found.

**FIGURE 2:**
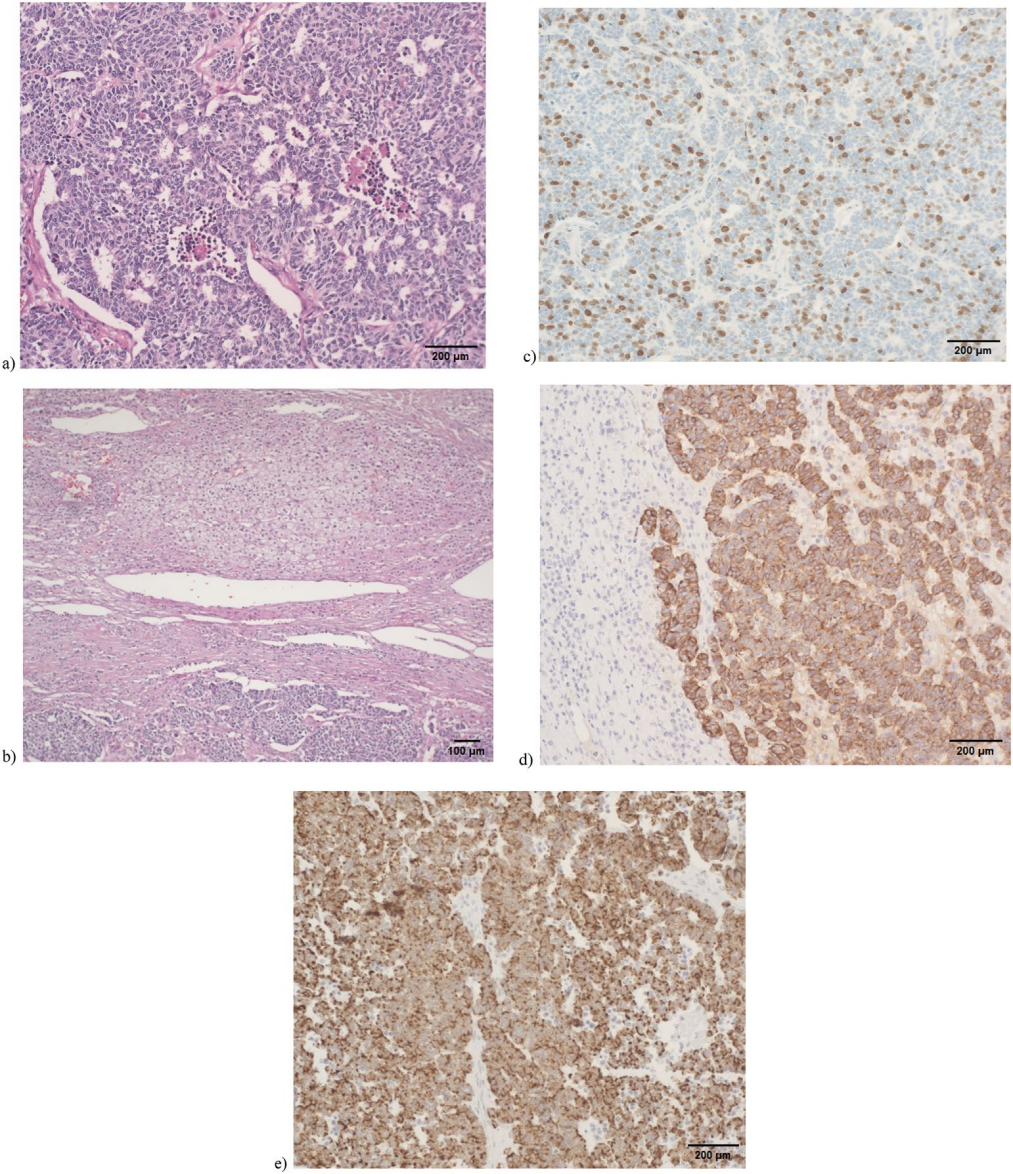
Cytopathology from core biopsy of left adrenal gland mass. (a) Tumor cells exhibit characteristic features of carcinoma. The tumor is composed of large neoplastic cells with necrotic foci (bar = 200 μm). (b) Adrenal cortex show carcinoma (bar = 100 μm). (c) The number of positively Ki‐67 stained nuclei in the tumor. The Ki‐67 index of proliferation was up to 25% (bar = 200 μm). (d) Tumor cells show positivity for chromogranin A (bar = 200 μm). (e) Tumor cells show positivity for cytokeratin (bar = 200 μm).

The specimen was initially misdiagnosed for malignant pheochromocytoma, with a mitotic index of 6/10 HPF. A second reading of the biopsy was performed and was in favor of a different final diagnosis of poorly differentiated SCNEC of the adrenal glands. The Ki‐67 labeling index showed a proliferation index of approximately 25%. Immunostains performed with adequate control showed that the neoplastic cells expressed cytokeratin, CK7, synaptophysin, and chromogranin, while they were negative for GATA3, TTF1, CDX2, and PAX8. These features were consistent with a diagnosis of high‐grade poorly differentiated NEC of the adrenal gland (Figure 2).

This second reading was performed upon the oncologist's request because the specimen was positive for chromogranin and cytokeratin which are markers of NET.

NETs generally show a local extension in 75% of cases with lymph node, bone, and liver involvement, and in 30% of patients, brain metastases are observed [1]. Total body CT and bone scans were negative for intrathoracic malignancy. Gastrointestinal endoscopy did not reveal any malignant lesions in the GI tract. Brain magnetic resonance imaging (MRI) showed an old ischemic stroke limited to the cortex and measuring 1.2 cm. Later on, an 18‐fluoro‐deoxy‐glucose positron emission tomography (FDG‐PET) CT scan will sum up all these negative findings, since it showed no distant systemic findings.

**FIGURE 3:**
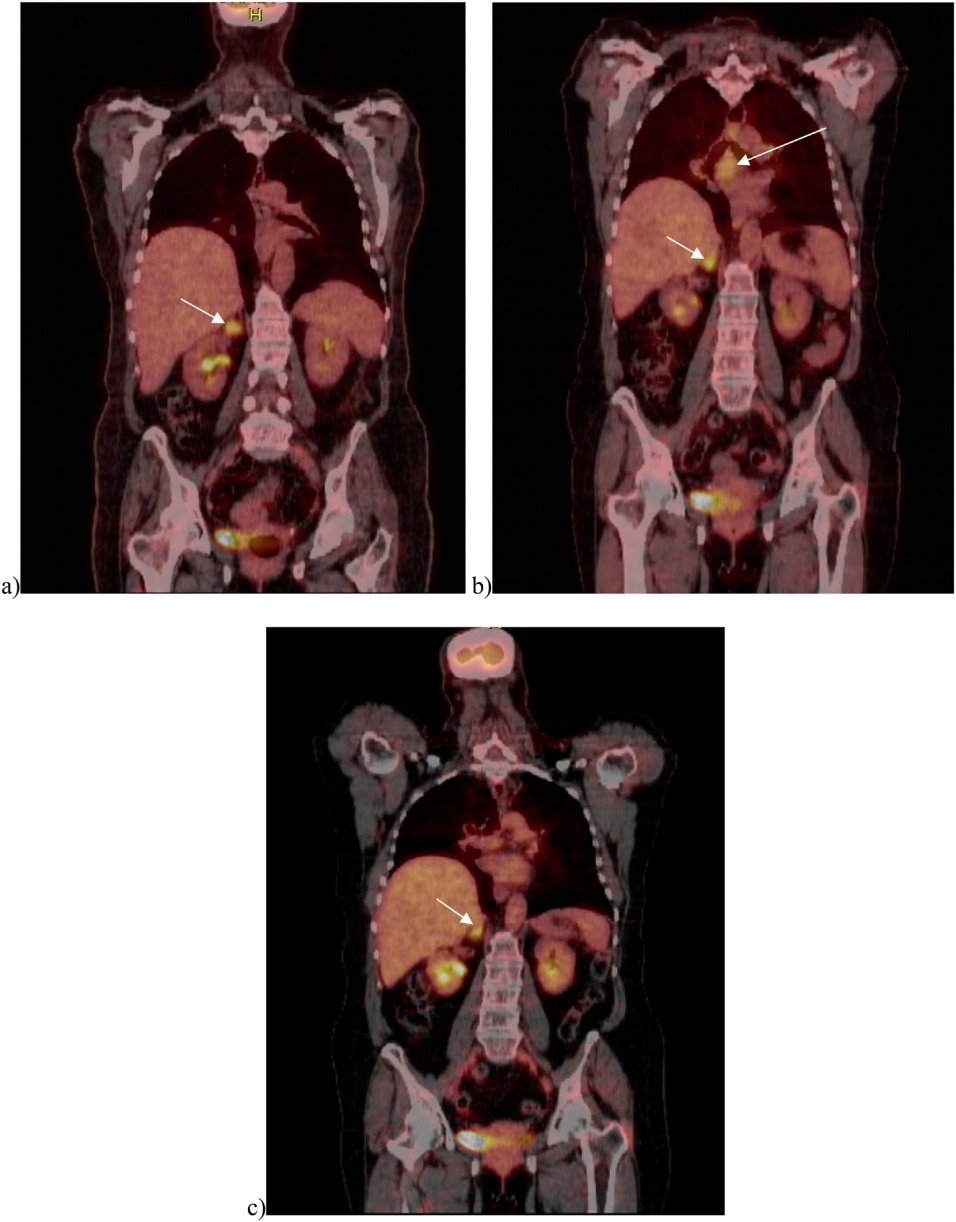
FDG‐PET/CT. (a) Initial FDG‐PET/CT revealed increased activity in the right adrenal mass with no additional site of uptake in coronal representation. (b) Follow‐up FDG‐PET/CT performed 3 months after the completion of immunotherapy showed a mild interval increase in the nodularity of the remaining part of the right adrenal gland with moderate uptake and interval increase in mediastinal nodal disease in coronal representation. (c) Follow‐up FDG‐PET/CT performed 3 months later showed complete resolution of mediastinal lymph nodes and a right adrenal showing normal physiological activity in coronal representation. No magnification of any of the images was done.

Primary high‐grade NEC are exceptionally confined to the adrenal glands. They are poorly understood and are mainly metastatic tumors with limited reports in the literature. When the diagnosis of SCNEC is made, a primary site should be meticulously searched for; therefore, medical oncology proposed an FDG‐PET CT to check for an extra‐adrenal primary. Possible primary sites include the lungs, GI tract, and pancreas. Other sites are scarcely ever affected but include the parathyroid, adrenal, and pituitary glands, and calcitonin‐producing cells of the thyroid [2, 5]. A staging FDG‐PET CT scan was performed. It revealed accumulation of 18‐FDG in the right adrenal gland and ruled out other sites of distant and locoregional metastases (Figure 3).

There were no other concerning regions of increased uptake to suggest an extra‐adrenal primary tumor, except for a 7 mm bleb in the right lower lobe that appeared on the CT scan and led to her final diagnosis (Figure 1).

This is most probably an oligometastatic NEC of unknown primary site with a burned‐out primary localized in her lungs and PNS causing her myopathy and algodystrophy [1, 6]. Even her hemolytic anemia seemed to be related to her disease. All of her symptoms could now be framed under one etiology. The patient then underwent an uncomplicated laparoscopic partial tumorectomy on her right adrenal gland, which revealed NEC as well. But this time the pathology report did not mention clean margins. The patient responded dramatically and rapidly to resection of both sites of the left adrenalectomy and contralateral partial adrenal tumor resection. The PNS, comprising algodystrophy and myopathy, resolved completely. She went from being a wheelchair bound with continuous uncontrollable pain to walking, and resuming a normal life, and her anemia resolved completely on its own.

We recommended then systemic therapy with adjuvant chemotherapy and immunotherapy, followed by focal low‐dose radiotherapy to the remnant right adrenal gland. Control whole‐body FDG‐PET and gadolinium‐enhanced brain MRI scans were performed every 6 months, initially showing no evidence of metastatic disease [7, 8, 9, 10].

In our case, the treatment guidelines were extrapolated from small‐cell lung cancer (SCLC) given the biological, histological, and cytological similarities.

In SCNEC of the lung, cisplatin and etoposide are considered gold standard treatment [11, 12, 13, 14]. We offered her to start combination chemoimmunotherapy as per the present recommendation of a small‐cell tumor, and she received cisplatin etoposide and Tecentriq combination. The patient completed four chemotherapy cycles. Immune checkpoint inhibitors have become a cornerstone in the treatment of cancer; therefore, Atezolizumab 1200 mg was added on day 1 and then continued as maintenance treatment Q3 weeks for several months because new data emerged that its addition benefits patients with metastatic SCLC [15]. The patient completed 17 maintenance doses of Atezolizumab.

Since the pathology report of the right adrenal did not mention clear margins, the patient was referred to a radiation oncologist one month after completion of chemotherapy. this radiayion oncologist was against administering radiotherapy since there are no published studies about adrenal tumors treated with both surgery and radiation. Finally, based on the medical oncology team's recommendations and on family's request, the patient ended up receiving five sessions of stereotactic body radiotherapy (SBRT) to the remnant right adrenal gland. Surveillance imaging performed 6 months later showed no evidence of recurrent disease.

A follow‐up FDG‐PET scan performed 3 months after the completion of a whole year of maintenance Atezolizumab showed new progressive lymphadenopathy in the left hilum of the lung reaching 1.5 cm and in the subcarinal region reaching 2.8 cm (Figure 3). In view of this, the differential diagnosis included tumor recurrence or an immune‐related adverse event (irAE) due to immunotherapy. With these novel agents, many new side effects have been reported, including irAEs which may mimic disease progression. Although disease relapse may cause mediastinal lymphadenopathy on chest imaging, an identical radiographic appearance is seen in irAEs caused by immunotherapy drugs [16].

Since we lacked a proper radiology reading and even though a biopsy is not routinely performed, it was performed in our case to rule out mediastinal relapse. EBUS results were negative for malignancy. These nodules represented reactive lymphadenopathies caused by the PD‐L1 inhibitor.

The PET scan also showed an active right adrenal gland with mild enlargement and an increase in its nodularity with moderate uptake. Therefore, a follow‐up PET scan was performed 3 months later to rule out any malignant process. Adrenal activity detected was most likely physiological, and no tumoral enlargement was seen (Figure 3).

Adrenal hypertrophy is thought to be related to a nonspecific response of the adrenal gland to stress, or as we presumed, compensatory to the loss of most other adrenal tissues. The imaging findings from our case are consistent with the diagnosis of acute radiation‐induced adrenal hypertrophy [17]. To our knowledge, this is the first case of bilateral adrenal gland tumors treated with unilateral adrenalectomy, right adrenal tumorectomy, and radiation, which show a compensatory hyperactivity in the remnant gland instead of adrenal insufficiency. Morning cortisol measured every 3 months for the entire year following radiotherapy continued to show levels at the normal upper limit.

**FIGURE 4:**
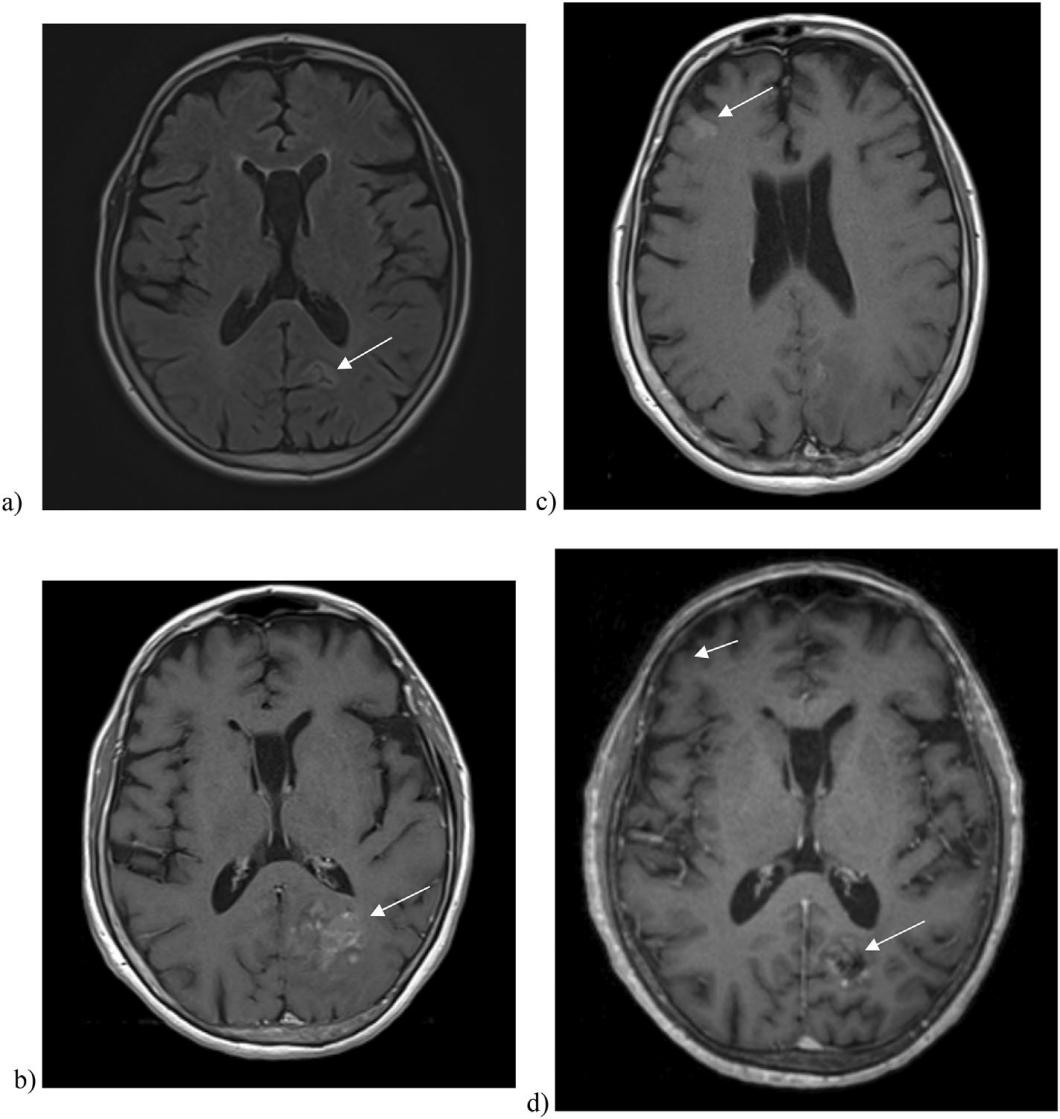
Brain MRI. (a) 1.2 cm lesion in the left inferior parietal lobe misinterpreted as a scar from an old stroke when first worked up in 2022 in axial representation. (b) Brain MRI performed 3 months after completion of an entire year of immunotherapy: a 3.3 × 2.8 cm lesion in the left inferior parietal lobe in axial representation. (c) Brain MRI performed 3 months after completion of an entire year of immunotherapy showed a small tumor measuring 1.5 cm in the right frontal lateral cortical area in axial representation. (d) Follow‐up brain MRI performed 3 months later: two brain lesions significantly decreased in size. Now measuring 2 cm versus 3 cm for the mass in the left inferior parietal lobe; and 4 × 8 mm versus 1.5 × 0.8 cm for the lesion in the right frontal lateral cortical area in axial representation. No magnification of any of the images was done. They were copied as they appeared on the viewer. The two images of the brain MRI labeled (b) and (c) were slightly modified by few millimeters in width to fit on the page.

Unfortunately, a follow‐up brain MRI revealed that the lesion in the left inferior parietal lobe was misinterpreted as a scar from an old ischemic stroke when first worked up at a peripheral hospital. In fact it was a brain metastases measuring initially 1.2 cm and was at present on repeat imaging measuring 3.3 × 2.8 cm, on top of an additional smaller tumor of 1.5 cm in the right frontal lateral cortical area (Figure 4). These two lesions helped to confirm our diagnoses since brain metastases are very infrequent in patients with extrapulmonary small‐cell carcinoma, and most NET patients with brain metastases show the primary lesion in the lungs [11, 18].

The two brain lesions that were still limited in size were addressed by the Medical and Radiation Oncology teams separately by stereotactic radiosurgery (SRS) in a total of six fractions. Of note, the patient had not received LD hippocampal‐sparing whole‐brain radiotherapy (WBRT) in view of her age and presentation, since the primary origin in the lung was never accepted by the radiologists.

An FDG‐PET scan performed 3 months later showed complete resolution of the mediastinal nodal disease, and no abnormal activity was detected anywhere in the body (Figure 3). Brain MRI showed major improvement in the previously seen masses. The tumors significantly decreased in size (Figure 4). For the time being, the patient remains on active surveillance with brain MRIs enhanced with gadolinium every 3 months and with FDG‐PET scan every 6 months. In this case, DOTA‐PET was not performed due to resource limitation. FDG‐PET remains a valuable tool for disease staging and prognosis. Further research is warranted to elucidate optimal follow up imaging in order to improve outcomes for this patient.

Tumor markers were not assessed in the blood workup because they would not serve alone in the follow up of this particular case especially since the patient is closely monitored every 6 months with and FDG‐PET scan. FDG‐PET scan is the ultimate monitoring tool of tumors of suspected lung origin and no additional complementary test is required when doing it on a regular basis.

## 3 Discussion

NETs can arise from neuroendocrine cells throughout the body and are commonly found in the respiratory and gastrointestinal tract. The diagnosis of NEC depends on histopathological recognition and reactivity to neuroendocrine markers such as synaptophysin, cytokeratin, and chromogranin A. The WHO classified tumor activity based on mitotic count and Ki‐67 proliferation index into NET G1, NET G2, NET G3, and NEC. NECs are histologically composed of either small or large cells.

The WHO classified NETs as well‐differentiated and poorly differentiated NETs.

The most common primary of NETs is in the gastrointestinal tract, the lungs, and the pancreas; other sites being seldom affected and usually considered metastatic tumors [7, 12].

The incidence of NEC of the adrenal glands is very uncommon, with fewer than a dozen cases reported in the literature (Table 1).

**TABLE 1:** Rare cases of NEC in adrenal glands.

Presentation	Cases (reference)	Percentage	Authors' name and year
Adrenal confined disease	[2]	9	Lee et al. 2020
	Unilateral [3, 4, 7, 8, 9, 10, 11, 12, 13]	82	Yamagata et al. 2022 Kanbara et al. 2017 Dong et al. 2013 Chang et al. 2014 Rahmani et al. 2021 Abdullah et al. 2023. Limonnik et al. 2020 Liu and Zhuang 2022 Ochiai et al. 2010
	Bilateral [5]	9	Ogawa et al. 2019
Metastases (organs involved)	Spleen [5]	9	Ogawa et al. 2019
	Kidney [7]	9	Dong et al. 2013
	Liver [12, 13]	18	Liu and Zhuang 2022 Ochiai et al. 2010
	Lung [9, 12]	18	Rahmani et al. 2021 Liu and Zhuang 2022
	Brain [3, 11]	18	Yamagata et al. 2022 Limonnik et al. 2020
	Bone [8]	9	Chang et al. 2014
	Lymph node [13]	9	Ochiai et al. 2010
	No metastases [2]	9	Lee et al. 2020
Chemotherapy (drugs) & immunotherapy	Cisplatin + Etoposide [2, 3, 8, 10, 11, 12]	55	Lee et al. 2020 Yamagata et al. 2022 Chang et al. 2014 Abdullah et al. 2023. Limonnik et al. 2020 Liu and Zhuang 2022
	Cisplatin + Campto [13]	9	Ochiai et al. 2010
	Amrubicin [3]	9	Yamagata et al. 2022
	Pembrolizumab + Bevazizumab [3]	9	Yamagata et al. 2022
	Atezolizumab [11]	9	Limonnik et al. 2020
	No chemo [5]	9	Ogawa et al. 2019
Radiotherapy	[3, 11]	18	Yamagata et al. 2022 Limonnik et al. 2020
Surgery	[2, 3, 4, 5, 8, 9, 11, 13]	73	Lee et al. 2020 Yamagata et al. 2022 Kanbara et al. 2017 Ogawa et al. 2019 Chang et al. 2014 Rahmani et al. 2021 Limonnik et al. 2020 Ochiai et al. 2010
Outcome	Stable [2, 3, 11, 13]	36	Lee et al. 2020 Yamagata et al. 2022 Limonnik et al. 2020 Ochiai et al. 2010
	Relapse [9]	9	Rahmani et al. 2021
	Died [5, 10, 12]	27	Ogawa et al. 2019 Abdullah et al. 2023 Liu and Zhuang 2022
	Loss of follow up [4, 7, 8]	27	Kanbara et al. 2017. Dong et al. 2013 Chang et al. 2014

The unknown etiology of NETs represents a challenge for their diagnosis and subsequent treatment. Due to the rarity of this specific type of neoplasm, there is no consensus among clinicians regarding its optimal management; therefore, different treatment strategies have been implemented to improve its prognosis. In our case, treatment was consistent with current recommendations for small‐cell tumors.

Adrenal NETs are rarely upfront bilateral or migrate to the brain. Brain and adrenal metastases are common in lung cancer patients. Approximately 20%–50% of brain metastases originate from lung cancer. Adrenal metastases from lung cancer can occur with a reported prevalence of 1.6%–3.5%. Thus, the brain and bilateral adrenal metastases in our case most probably originated from a lung cancer [18].

The 7 mm bleb that was found in her right lobe must have been the primary tumor that was burned out by her immune system, reason for which she may have developed brain metastases.

Moreover, this disease must have been immunogenic since it was self‐controlled by producing auto‐antibodies to which the primary resolved in total while the metastases were kept in check and not allowing additional spread. Moreover, they were responsible for the patient's dual PNS, leading to hemolytic anemia and causing the patient to become disabled. An extremely rare and unusual case.

PNSs are rare disorders with complex systemic clinical manifestations due to underlying malignancies. Researchers believe that PNSs are caused by the cancer‐fighting abilities of the immune system, specifically antibodies and T‐cells. These immune system agents attack not only cancerous cells, but also normal cells of the nervous system, causing neurological disorders. These syndromes may affect any part of the nervous system, from the cerebral cortex to the neuromuscular junction and muscle, damaging one or multiple areas. They can occur with or without detectable auto‐antibodies in serum and cerebrospinal fluid. The precise incidence and prevalence of PNSs are unknown because of the rarity of this condition. Neuropathies are the most common presentations of these neurological manifestations.

Autoimmune hemolytic anemia (AIHA) is a rare PNS associated with malignant solid tumors. The mechanisms underlying PNS‐AIHA are not yet well understood. We hypothesized that our patient's initial presentation of AIHA was a paraneoplastic phenomenon associated with her underlying malignancy. Successful treatment of the malignancy is the key to managing PNS. In our case, the hemolytic anemia resolved completely on its own after surgery.

PNS is a diagnosis of exclusion, and all possible etiologies should first be ruled out. The usual culprit being SCLC [6].

## 4 Conclusion

Sparse literature exists for adrenal gland NEC; therefore, there is a lack of standard treatment guidelines. This case highlights the complexities in the workup and management of adrenal masses. The origin of these malignancies is not yet completely understood, as a healthy adrenal gland does not contain neuroendocrine cells.

Multimodal management should be considered, emphasizing the need for individualized approaches. In the present case, the patient's symptoms and condition improved after adrenalectomy, chemotherapy, and radiotherapy. Thus, when metastatic lesions with unknown primary tumors are confirmed by pathological examination or even clinically diagnosed by imaging, clinicians should consider aggressive therapeutic options and investigate the primary tumor site [9, 12, 18].

Surgical elimination of oligometastases can offer long‐term disease control as part of a multimodal approach. Therefore, the primary treatment modality should be radical surgery when possible and may be curative; thus, early diagnosis is paramount. Followed by systemic chemotherapy, which plays an indispensable role in the therapeutic algorithm.

Our case represents a possible treatment approach that may provide better clinical outcomes.

## Author Contributions

**Carla J. El Hajj Mouawad:** conceptualization, data curation, formal analysis, project administration, visualization, writing – original draft. **Francois Georges Kamar:** conceptualization, data curation, formal analysis, investigation, supervision, validation, visualization, writing – review and editing.

## Ethics Statement

Ethical approval is not required for this study in accordance with local or national guidelines.

## Consent

Written informed consent was obtained from the patient for publication of this case report and accompanying images.

## Conflicts of Interest

The authors declare no conflicts of interest.

## Data Availability

The data that supports the findings of this study are available in the Supporting Information of this article.
